# A Rare Case Report of Mid Cavitary Takotsubo: The Role of Magnetic Resonance Imaging

**DOI:** 10.1155/2011/481394

**Published:** 2011-07-27

**Authors:** Jesse Sherratt, Carolyn E. McDonald, Gerald York, Ahmad Slim

**Affiliations:** ^1^Cardiology Service, Brooke Army Medical Center, Fort Sam Houston, San Antonio, TX 78234, USA; ^2^Cardiology Service MCHE-MDC, Brooke Army Medical Center, 3851 Roger Brooke Drive, San Antonio, TX 78234-6200, USA

## Abstract

This is a case report of a female presenting originally with clinical picture of acute coronary syndrome and depressed left ventricular function with no angiographic evidence of coronary artery disease with mid cavitary akinesis and basal as well as apical hyperkinesis after emotional stresses identified by multi-imaging modalities to be mid cavitary Takotsubo. The Incidence and the prevalence of apical ballooning syndrome (Takotsubo) is on the rise with more reports in the literature; however, mid cavitary Takotsubo remains rare and raises questions more than answers as to the reason behind the mid cavitary localization in some patients versus apical involvement.

## 1. Case Report

**Figure 1:**
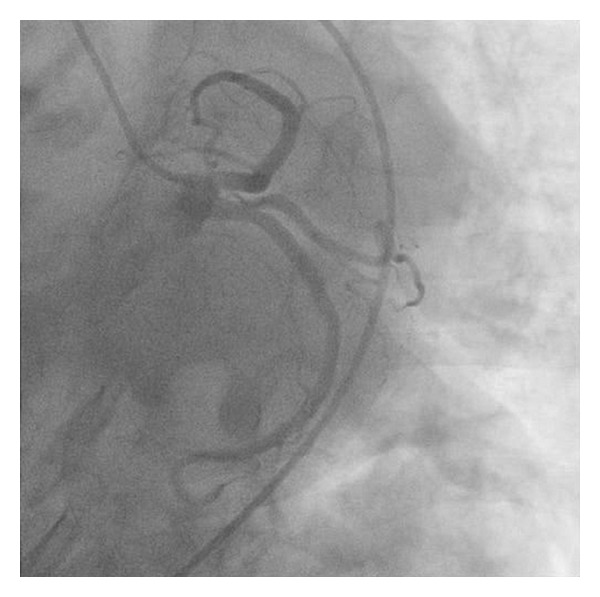
Coronary angiography of the left coronary tree with no angiographic evidence of obstructive disease.

**Figure 2:**
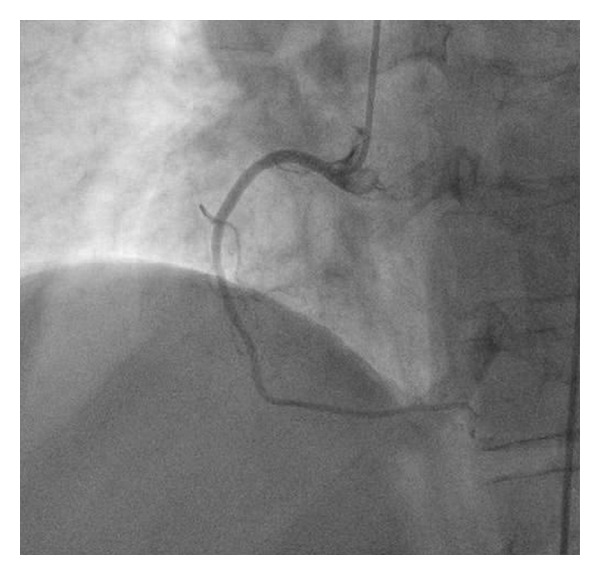
Coronary angiography of the right coronary tree with no angiographic evidence of obstructive disease.

**Figure 3:**
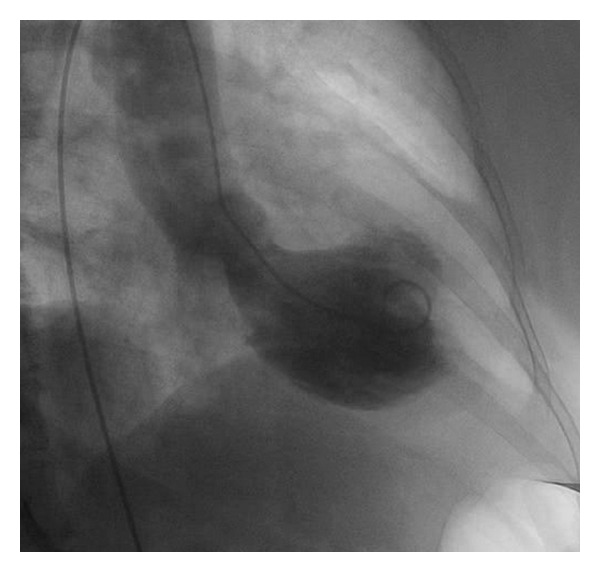
Left ventriculogram showing apical and basal hyperkinesis and mid cavitary akinesis.

**Figure 4:**
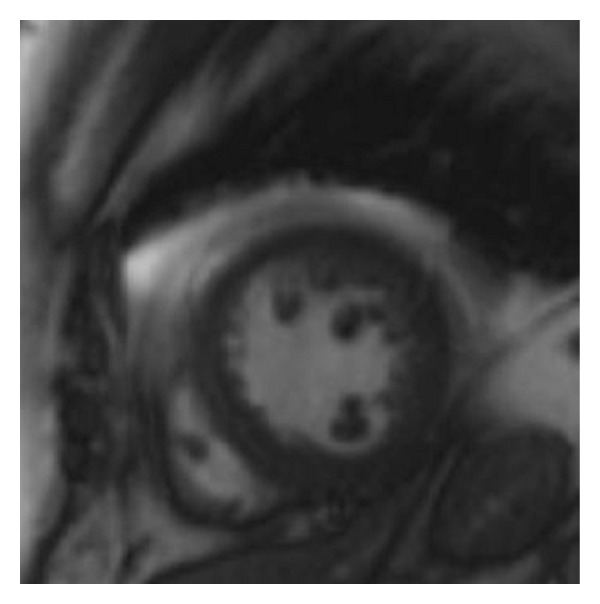
Mid cavitary view of the left and right ventricle on cardiac MRI.

This is the case of a 69-year-old obese female with history significant for hypertension and hyperlipidemia presenting with midsternal chest pain with radiation to right arm, right jaw, and dyspnea. Physical exam was unremarkable. Her initial complete blood count, chemistry panel, and TSH, as well as coagulation panel were normal with the exception of her cardiac enzymes (CK 248 IU/L, CK-MB 32.6 ng/mL, Troponin I 1.18 ng/mL (normal <0.04)). Patient was diagnosed with NSTEMI and admitted to cardiology critical care service and was treated overnight with dual antiplatelet therapy, anticoagulation, beta blocker, and ACE inhibitor, as well as a statin. Cardiac enzymes continued to downtrend overnight and patient remained chest pain free. Patient underwent left heart catheterization early morning that revealed no angiographic evidence of disease in all epicardial coronaries with evidence of mid cavitary akinesis and apical as well as basal hyperkinesis with ejection fraction (EF) calculated at 37% and mildly elevated left ventricular end-diastolic pressure (Figures 1, 2, and 3). Afterwards, patient admitted to emotional stresses in her life that peaked 48 hours prior to presentation. All above medications were discontinued at this point except for the beta blocker and the ACE inhibitor. Transthoracic echo performed the same day revealed severe mid cavitary hypokinesis with EF estimated to be 35–40% and mild mitral regurgitation. Cardiac magnetic resonance imaging was performed the next day and revealed no areas of enhancement within the epicardium, the myocardium, or the endocardium ruling out myocarditis or infarct with mid cavitary hypokinesis and EF estimated to be 38% (Figure 4). Patient was diagnosed with mid cavitary Takotsubo syndrome based on clinical presentation, elevated biomarkers without angiographic evidence of coronary artery disease or spasm on angiography, new onset cardiac wall motion abnormalities, and no areas of enhancement on MRI, resulting from recent extreme emotional stresses. This case is unique as the location of dyskinetic and akinetic myocardium was not apical but rather mid cavitary. Patient started on selective serotonin receptor blocker as recommended for diagnosis of Takotsubo in addition to maximizing medical management of her acutely decreased EF with beta blockade, ace inhibitor, and aspirin. Patient discharged home in stable condition with consequent followup showing complete recovery of her left ventricular systolic function.

## 2. Discussion

The syndrome of Takotsubo cardiomyopathy (or apical ballooning syndrome) is rare and has only been described in 1–2.2% of hospital admissions for acute coronary syndrome [1]. Mid cavitary dyskinesia and ballooning is a unique presentation of this already uncommon disease. To the best of our knowledge, only a few case reports exist that show mid cavitary involvement with the majority of studies concerning the clinical findings and pathophysiology of this disease being reported with apical ballooning only. The patient herself was typical of those in the other studies as the majority of those involved were postmenopausal and had recently suffered significant emotional stress [2]. Her presentation was also typical in that she presented with midsternal chest pain and elevated cardiac biomarkers. Of note, both a prior left heart catheterization and myocardial perfusion scan showed a preserved EF with no wall motion abnormalities. Currently, precise magnetic resonance imaging (MRI) data are not yet available and there is little evidence for the differential diagnosis of apical ballooning syndrome assessed by MRI [3]. A study by Eitel et al. in the European Heart Journal proposed that cardiac MRI can add valuable information in all patients with suspected apical ballooning syndrome for further differential diagnosis such as myocarditis, myocardial infarction, and coronary emboli with spontaneous lysis [3]. Eitel et al. found that typically delayed enhancement due to myocardial infarction shows subendocardial or transmural enhancement. However, delayed enhancement is not specific for myocardial infarction and can occur in a variety of other disorders such as myocarditis [3]. Their findings were consistent with those of others that found in patients with apical ballooning syndrome no pathologic signal activity can be documented in late enhancement imaging excluding myocardial infarction or myocarditis [3–6]. The lack of this delayed enhancement demonstrates that the area of apical ballooning does not undergo irreversible damage, thus, explaining the recovery of systolic function in patients with apical as well as mid cavitary Takotsubo as seen in our patient. While MRI has more commonly been studied in apical ballooning syndrome, it appears to be as useful in discerning potential mid cavitary involvement and could be considered as a confirmatory test.

## 3. Conclusion

To our knowledge, this is one of the few unique cases reported of mid cavitary Takotsubo as confirmed by left heart catheterization, and transthoracic echo as well as cardiac magnetic resonance imaging. Patient is symptom free on optimal medical therapy and will be longitudinally followed up with serial echocardiography and physical exam to document resolution. As cardiologists are becoming more and more aggressive in early invasive angiography for risk stratification, and more cases of Takotsubo are reported, we do anticipate that the prevalence of this condition will be higher than anticipated.
